# Impact of caste on the neurodevelopment of young children from birth to 36 months of age: a birth cohort study in Chitwan Valley, Nepal

**DOI:** 10.1186/1471-2431-14-56

**Published:** 2014-02-27

**Authors:** Rajendra Prasad Parajuli, Takeo Fujiwara, Masahiro Umezaki, Chiho Watanabe

**Affiliations:** 1 Department of Social Medicine, National Research Institute for Child Health and Development, 2-10-1 Okura, Setagaya-ku, 157-8535 Tokyo, Japan; 2 Department of Human Ecology, Graduate School of Medicine, University of Tokyo, 7-3-1 Hongo, Bunkyo–ku, 113-0033 Tokyo, Japan

**Keywords:** Nepal, Caste system, Socioeconomic position, Child development, Toxic elements, Essential elements

## Abstract

**Background:**

Caste, a proxy of socioeconomic position, can influence the neurodevelopment of children through several pathways, including exposure to toxic elements. Studies from developing countries where caste is represented by prevailing caste groups and people are highly exposed to toxic elements can provide useful insights into the mechanisms of neurodevelopmental inequities among children. This study aims to investigate the impact of caste on the neurodevelopment of children from birth to 36 months of age in Chitwan Valley, Nepal, where people are exposed to high levels of arsenic (As) and lead (Pb).

**Methods:**

Participants (N = 94) were mother-infant pairs from the Chitwan district in Nepal. The neurodevelopment of the infants was assessed using the Brazelton Neonatal Behavioral Assessment Scale, Third Edition, (NBAS III) at birth and the Bayley Scales of Infant Development, Second Edition, (BSID II) at ages 6, 24, and 36 months. Caste was categorized based on surname, which, in Nepal generally refers to one of four caste groups. We also measured the concentrations of As and Pb in cord blood.

**Results:**

Caste was positively associated with the state regulation cluster score of the NBAS III at birth after adjustment for covariates (p for trend < 0.01). Adding cord blood As levels attenuated the association (p for trend = 0.12). With regard to neurodevelopment at six months of age, the third-ranked caste group scored higher than the first-ranked caste group on the Mental Development Index (MDI) of the BSID II (coefficient = 3.7; 95% confidence interval (CI) = 1.3 to 6.0). This difference remained significant after adjustment for cord blood As levels and other covariates was made (coefficient = 3.9; 95% CI = 1.2 to 6.7). The remaining clusters of the NBAS III and BSID II at 6, 24, and 36 months were not significantly associated with caste group.

**Conclusions:**

Caste was positively associated with the state regulation cluster score of NBAS III at birth. This association was partially mediated by cord blood As levels. However, the negative impact of caste on neurodevelopment disappeared as the children grew. Furthermore, an inverse association between caste and MDI at six months of age was observed. Additional studies are needed to elucidate the mechanism of how caste affects neurodevelopment.

## Background

Exposure to socioeconomic disadvantage during pregnancy and early childhood impairs neurodevelopment in children [1,2]. Despite evidence indicating that the association between socioeconomic position (SEP) and the neurodevelopment of newborns and young children [3-9], the results of epidemiological studies on the association of SEP with later neurodevelopment have been inconsistent [6,7,10].

For example, in the Port Pirie prospective cohort study, Tong et al. [7] reported a 0.8–2.0 unit increment in children’s cognitive scores per ten-unit increment in their SEP scores, while in a Bolivian cohort, Ruiz-Castell et al. [10] could not detect an association between SEP and cognitive development. Such discrepancies may have been caused by differential representation of actual SEP by the assessment indicators used. In the Bolivian cohort, most of the participating families did not have any members with permanent jobs (rather, they held temporary, short-term jobs); thus, parents’ occupation (which was used as a proxy of SEP) likely did not reflect actual social or economic level. However, although SEP was evaluated by a similar indicator (i.e., parents’ occupation) in the Port Pirie cohort, the fact that they held professional (permanent) occupations may have reflected their actual social or economic level. Other studies have considered family income, education, and occupation as proxies of SEP [6,11-13]. However, the use of these proxies might not exactly represent SEP as such, because each proxy measures a different aspect of SEP [14]. Considering these disadvantages of SEP measurement, caste group might be a good indicator of SEP. To the best of our knowledge, no previous studies have evaluated the effect of caste group on neurodevelopment in young children.

Caste refers to a person’s status within the structure of society. In Nepal, the caste system still forms an important pillar of the social hierarchy [15], even though it was officially abolished by law in 1964 [16]. The Hindu caste structure segregates people into four caste groups on the bases of ritual purity and occupation, namely *Brahmin*, *Chetri*, *Vaishya*, and *Shudra*. The *Brahmins*—who taught, interpreted religious customs and rules, and administered the Hindu religion—were at the top of the hierarchy. They were followed by the *Chetri*, who were considered the rulers and warriors of society. Next in the hierarchy were the *Vaishya*, who were farmers and merchants. At the lowest level were the *Shudra,* who were laborers made to serve those belonging to the upper three castes [17].

We hypothesized that caste group is associated with neurodevelopment in young children through exposure to toxic elements during pregnancy. Toxic elements, such as lead (Pb) or arsenic (As), are harmful to neurodevelopment because they can induce oxidative stress and the production of free radicals, resulting in neuronal apoptosis [18,19]. We targeted Chitwan Valley in lowland Nepal because this district is exposed to high levels of As via high-level As contamination [20]. In addition, Pb exposure is high in this district because the region is situated at the junction between two main highways from Kathmandu and East-West Highway; this location serves as a major artery for a number of vehicles that emit Pb into the environment via leaded gasoline [21]. Further, this district is well recognized as a central immigration target among many caste groups from different parts of the country [22]. Thus, it was hypothesized that the association between neurodevelopment and caste group via exposure to toxic elements would be more visible in the Chitwan district versus others in Nepal. The objectives of the present study were to investigate the impact of caste on neurodevelopment scores from birth to three years of age, and to investigate whether it is driven by exposure to toxic elements during pregnancy in the Chitwan District.

## Methods

### Study sample

The eligibility criteria to be met for participation in the present study were as follows: living in the Chitwan Valley for at least two years, full term pregnancy (i.e., more than 37 weeks of gestation) at a specified hospital visit, aged 18–40 years, *per vaginam* delivery, singleton birth, and no reports of diabetes, hypertension or pre-eclampsia. Two hundred pregnant mothers were approached from September to October 2008 in the Bharatpur General Hospital of the Chitwan district. Among them, 119 were eligible to participate in the study. Eligible mothers were informed of the background and objectives of the study, what they would experience during the study process, the potential benefits they might experience and potential (although unexpected) risks. One hundred women (84%) signed a letter of informed consent and participated. The study protocol was approved by the Ethical Committee of the Graduate School of Medicine at the University of Tokyo (approval no. 2244) and that of the Bharatpur General Hospital, Chitwan, Nepal.

### Neurodevelopmental indicators

The third edition of the Brazelton Neonatal Behavioral Assessment Scale (NBAS III) [23] was used to assess neurodevelopment at birth. The NBAS III has frequently been used in the field of neurotoxicology [24,25]. Details regarding NBAS III assessments and research findings from this cohort have been published previously [26]. NBAS III clusters were composed of 7 dimensions: habituation, orientation, motor system, state organization, state regulation, autonomic stability, and abnormal reflex.

The second edition of the Bayley Scales of Infant Development (BSID II) [27] was used to assess neurodevelopmental status at ages 6, 24 and 36 months. The BSID II scale has also frequently been used in the field of neurotoxicology [28,29]. The BSID II provides three neurodevelopmental clusters: Mental Development Index (MDI), Psychomotor Development Index (PDI), and Behavioral Rating Scale (BRS). The MDI reflects an infant’s level of cognitive function, language, and personal and social development. The PDI reflects gross and fine motor function, and the BRS is a record of the examiner’s impression of the infant’s neurobehavioral integrity.

The BSID II test was conducted in the infants’ homes and administered within four weeks of the target age (i.e., at age 6, 24 or 36 months ± one month). The infant’s age in number of days at the time of testing was also recorded. Single rater (RPP) conducted both the NBAS III and BSID II assessment.

### Caste groups

During the interview process, the complete name, detailed home address, and mobile or home phone number of the parents was collected to enable the collection of prospective follow-up data. There is no single widely accepted definition or classification of each caste group [30]. Thus, we classified participants into the four ancient caste groups according to occupational specialization (i.e., *Brahmin* [priest], *Chetri* [warrior], *Vaishya* [trader and farmer], and *Shudra* [laborer]) [31]. These groupings are widely accepted despite the formal abolition of the caste system. In the present study, the classification was based on the surname of the father of the cohort baby (see Table 1 for details). However, in cases of confusing surnames, phone conversations were held with parents to ascertain their caste groups. The rank of the caste group was placed in the following order: *Brahmin, Chetri*, *Vaishya*, *and Shudra* were ranked first, second, third, and fourth respectively*.*

**Table 1:** Categorization of caste groups according to surname

**Caste name**	**Surname or Family name**
*Brahmin*	Kharel, Adhikari, Subedi, Kandel, Chapagain, Gaire, Dhakal, Neupane, Sharma*, Gautam, Sapkota, Rijal, Dawadi, Neure, Kattel, Khanal, Parajuli, Wosti, Pathak, Puri, Nepal, Poudel, Aryal, Lamichhane, Bhattarai, Prasai, Ghimire*, Shinkhada,
*Chetri*	*Thapa, Chhetri, Kuwar, Khadka, Khatri, KC, Thakuri, Burlakoti, Yadav*
*Vaishya*	*Chaudhari, Shrestha, Mahato, Manandhar, Rauniyar, Lama, Tamang, Aale, Gurung, Thapa*
*Shudra*	Pariyar, BK, Sunar, Kumal, Giri*, Sharma*, Baraili, Nagarkoti, Rana, Darai

*The same surname is currently being used by *Shudra* for social assimilation. However, they were categorized into four caste groups based on home and phone consultations.

### Measurements of cord blood toxic elements levels

To assess the levels of toxic elements, cord blood was collected from the placenta by midwives following common aseptic procedures. Cord blood (10 mL) was collected into a trace-metal–free cryovial that contained ethylene diamine tetra-acetic acid (EDTA) as an anticoagulant. Cord blood samples were stored in a standard freezer (-20°C) for less than one month, then kept frozen with dry ice during transport to a laboratory in Tokyo where they were stored in a deep freezer (-78°C) until analyzed. Detailed methods regarding the measurement of cord blood As and Pb levels in this cohort have been published previously [26].

### Covariates

The height and weight of mothers were recorded before delivery. Body weight was measured to the nearest 0.1 kg using a portable digital scale (Model BF-046 WH; Tanita, Tokyo, Japan). Height was measured to the nearest 0.1 cm. Body mass index (BMI) was calculated by dividing weight (kg) by height squared (m^2^). The birth weights of the newborns were obtained from hospital records. Height and weight were also taken at 6, 24, and 36 months of age using the same devices and methods.

The following information was collected during hospital face-to-face interviews via a structured questionnaire: mother’s age, mother’s parity, baby’s gender, gestational age, time and date of delivery, mother’s level of education, annual family income, mother’s smoking status during pregnancy, and mother’s status of alcohol intake during pregnancy. A single rater (RPP) visited the home of each mother-infant pair approximately 6 and 36 months after delivery and evaluated the postnatal home environment on the Home Observation for Measurement of Environment scale (HOME) [32].

### Statistical analyses

The distribution of all variables were examined for normality. Cord blood levels of toxic elements and annual family income were log transformed. Associations between caste group and demographics, birth outcomes, and prenatal and postnatal environmental variables were analyzed by linear trend tests.

Using a bivariate model, the individual associations between caste group and each NBAS III cluster score were analyzed. Multivariate analyses were conducted and adjusted for mother’s age [33], parity [29], family income [34], mother’s level of education [29,35], mother’s BMI before giving birth [36], gestational age [36] and infant’s age at the time of the NBAS III assessment (Model 1). Further, As levels were adjusted (Model 2). For ages 6, 24, and 36 months, multiple regression models of each BSID II cluster, MDI, PDI, and BRS were adjusted for maternal age, maternal education, log-transformed income, parity, maternal BMI, birth weight, concurrent age at BSID assessment, infant weight, and HOME score (Model 1) and log As levels was further adjusted (Model 2). The 6-month-old HOME score was adjusted to create the model for 24-months-old since the 6-month score can represent the home environment for up to three years. Trend tests for neurodevelopment indicators were also performed by caste group. Statistical significance was determined with a criterion level set at *p* < 0.05. All analyses were performed with a statistical software package (Stata version 11.0).

## Results

Table 2 summarizes the characteristics of mother-infant pairs at birth and at 6, 24, and 36 months after birth. The maternal, household, and newborn characteristics of this cohort have been published previously [26]. The mothers’ level of education showed a linear trend in terms of caste (i.e., more education among higher-ranked caste, *p* < 0.001). Infants’ ages during the six-month BSID II assessment also varied by caste rank (*p* = 0.02). Evaluation using the BSID II began in areas distant from the Chitwan Valley, where predominantly lower-caste parents reside. Thus, babies’ ages at BSID II assessment were lower among lower-caste groups. The HOME scores were consistently higher among high-ranked caste groups than low-ranked caste groups at ages 6 and 36 months (*p* < 0.001).

**Table 2:** Characteristics of participants in a birth cohort study: at birth, 6, 24 and 36 months after birth

	**Caste groups, Mean (SD) or N (%)**				***P *** **for trend**
	***Brahmin***	***Chetri***	***Vaishya***	***Shudra***	
	**(N = 37, 39.4%)**	**(N = 13, 13.8%)**	**(N = 26, 27.7%)**	**(N = 18, 19.2%)**	
Mothers characteristics at birth (n = 94)					
Age (years)	23.4 (3.1)	22.4 (2.5)	23.1 (4.4)	22.3 (4.5)	0.41
Primipara n (%)	26 (27.7)	11 (11.7)	13 (13.8)	12 (12.8)	0.31
Education level (median, years)	**12**	**10**	**8**	**8**	**<0.001**
BMI (kg/m^2^)	23.3 (2.5)	22.9 (2.3)	22.7 (3.8)	24.1 (2.9)	0.6
Newborn babies characteristics (n = 94)					
Gestational age (weeks)	39.1 (1.4)	39.5 (1.7)	39.1 (1.3)	38.8 (0.9)	0.61
Sex of baby n (% male)	19 (51.4)	7 (54.0)	11 (42.3)	8 (44.4)	0.48
Age of neurodevelopmental assessment					
NBAS III at birth (in hours) (n = 94)	17.2 (4.0)	17.1 (2.9)	17.7 (3.0)	17.6 (2.5)	0.56
BSID II assessment at 6 months (in days) (n = 94)	**195.3 (13.2)**	**199.2 (12.7)**	**188.7 (12.3)**	**188.9 (12.6)**	**0.02**
BSID II assessment at 24 months (in months) (n = 89)	25.9 (0.4)	25.9 (0.4)	25.7 (0.4)	25.9 (0.4)	0.36
BSID II assessment at 36 months (in months) (n = 83)	36.9 (0.4)	36.9 (0.3)	36.7 (0.4)	36.9 (0.4)	0.37
Household characteristics					
Annual family income (USD)	2891 (3256)	1944 (1697)	2798 (3434)	1771 (1744)	0.06
Total HOME Scale score at 6 months (n = 94)	**31.8 (5.4)**	**30.3 (4.0)**	**26.8 (4.7)**	**26.5 (4.3)**	**<0.001**
Total HOME Scale score at 36 months (n = 83)	**41.2 (6.0)**	**40.0 (6.0)**	**36.5 (6.8)**	**33.9 (7.6)**	**<0.001**

Bold signifies p < 0.05.

Table 3 summarizes the distribution of cord blood Pb and As levels, and birth outcomes according to caste group at birth and 6, 24, and 36 months after birth. Higher cord blood levels of As were found among lower-ranked caste groups (*p* < 0.01), but Pb was not significantly associated with caste (*p* = 0.59). Birth weight and body weight at ages 6, 24, and 36 months did not differ by caste group. Among the NBAS III clusters, the newborns’ regulation of state cluster score (as evaluated by the NBAS III) was more elevated in higher-caste groups (*p* < 0.01). The remaining clusters of the NBAS III were not associated with caste group. Scores on the BSID II indices (i.e., MDI, PDI, and BRS) did not differ by caste group at ages 6, 24, or 36 months.

**Table 3:** Distribution of birth outcome variables by caste groups in a birth cohort study: at birth, 6, 24 and 36 months after birth

	**Caste groups, Mean (SD) or Median**				***P *** **for trend**
	***Brahmin***	***Chetri***	***Vaishya***	***Shudra***	
	**(N = 37)**	**(N = 13)**	**(N = 26)**	**(N = 18)**	
*In utero* exposure of toxic elements (n = 94), mean					
Arsenic (μg/L)§	**1.3 (1.4)**	**1.3 (0.3)**	**1.7 (0.7)**	**1.5 (0.5)**	**<0.01**
Lead (μg/L)§	30.5 (40.3)	31.4 (27.7)	36.7 (39.6)	26.4 (17.5)	0.59
Anthropometric characteristics, mean					
Birth weight (kg) (n = 94)	3.0 (0.5)	3.0 (0.4)	3.1 (0.4)	3.1 (0.5)	0.16
Body weight (kg) at 6 months after birth (n = 94)	7.3 (1.1)	7.3 (0.8)	7.3 (0.7)	7.3 (0.8)	0.93
Body weight (kg) at 24 months after birth (n = 89)	11.5 (1.6)	11.0 (1.3)	10.9 (1.2)	10.8 (1.0)	0.09
Body weight (kg) at 36 months after birth (n = 83)	12.9 (1.7)	12.5 (1.1)	12.4 (1.4)	12.6 (1.3)	0.30
NBAS III cluster score at birth (n = 94), median					
Habituation	27	27	28	26.5	0.47
Orientation	45	37	44	38	0.30
Motor system	26	24	25.5	26	0.21
State organization	11	14	15.5	9.5	0.58
State regulation	**28**	**28**	**25**	**26**	**<0.01**
Autonomic stability	13	13	13	13	0.26
Abnormal reflex	7	7	5	5	0.23
BSID II cluster score at 6 months after birth (n = 94), median					
MDI	104	104	107	104	0.07
PDI	103	100	101.5	104	0.80
BRS	122	127	124	121	0.39
BSID II cluster score at 24 months after birth (n = 89), median					
MDI	94	94	94	92	0.49
PDI	100	107	104.5	100	0.75
BRS	106	114	109.5	104	0.43
BSID II cluster score at 36 months after birth (n = 83), median					
MDI	95	93	97	90	0.19
PDI	116	116	119	116	0.60
BRS	106	104	102	104	0.17

§Log transformed values were tested.

Bold signifies p < 0.05.

NBAS: Brazelton neonatal behavioral assessment scale, BSID: Bayley scale of infant development, MDI: mental development index, PDI: psychomotor development index, BRS: behavioral rating scale.

Table 4 shows the coefficients of NBAS III clusters at birth by caste group with reference to *Brahmin*, the first-ranked caste group. In Model 1—which was adjusted for maternal age and education, log-transformed income, maternal BMI, age at NBAS III assessment, parity, and birth weight—the state regulation cluster score for *Vaishya* (i.e., the third-ranked caste group) was lower than the score for *Brahmin* (coefficient = –3.6; 95% CI = –5.8 to –1.3). While attenuated, the association remained significant in Model 2 when including log As as a covariate, (coefficient = –2.8; 95% CI = –5.3 to –0.3). Although the trend was significant in Model 1, it became insignificant in Model 2, suggesting that cord blood As levels mediated the association between caste group and state regulation score at birth. Interestingly, significantly higher scores in the state organization cluster were found among the third-ranked caste group in Model 1, however, the corresponding association was not significant in Model 2. The remaining NBAS III cluster scores were not associated with caste group.

**Table 4:** Coefficient and 95% confidence interval of social status or caste group with NBAS III clusters at birth using multivariate regression model (n = 94)

		***Brahmin***	***Chetri***	***Vaishya***	***Shudra***	**p for trend**	**Constant**
		**(N = 37)**	**(N = 13)**	**(N = 26)**	**(N = 18)**		
Habituation	Crude	Ref	0.4 (-1.8 to 2.5)	1.6 (-0.1 to 3.3)	-0.1 (-2.0 to 1.9)	0.47	26.4
	Model 1	Ref	0.5 (-1.9 to 2.8)	1.9 (-0.1 to 3.9)	-0.0 (-2.4 to 2.3)	0.51	27.4
	Model 2	Ref	0.4 (-1.9 to 2.8)	1.8 (-0.3 to 3.8)	-0.1 (-2.5 to 2.3)	0.62	27.2
Orientation	Crude	Ref	-5.7 (-12.4 to 1.0)	0.5 (-4.8 to 5.8)	-4.9 (-10.9 to 1.1)	0.30	42.1
	Model 1	Ref	-5.5 (-12.6 to 1.6)	-0.2 (-6.3 to 5.9)	-6.3 (-13.5 to 0.9)	0.23	27.4
	Model 2	Ref	-5.4 (-12.5 to 1.7)	0.3 (-6.0 to 6.7)	-6.0 (-13.3 to 1.3)	0.27	27.7
Motor system	Crude	Ref	0.7 (-2.6 to 1.1)	0.2 (-1.3 to 1.6)	1.1 (-0.5 to 2.8)	0.21	24.5
	Model 1	Ref	-0.5 (-2.5 to 1.4)	0.3 (-1.4 to 2.0)	1.4 (-0.6 to 3.4)	0.20	26.6
	Model 2	Ref	-0.5 (-2.4 to 1.5)	0.8 (-0.9 to 2.5)	1.7 (-0.3 to 3.7)	0.09	27.2
State organization	Crude	Ref	0.7 (-2.6 to 4.0)	1.9 (-0.7 to 4.5)	-2.1 (-5.0 to 0.8)	0.59	13.5
	Model 1	Ref	1.7 (-1.7 to 5.2)	**3.1 (0.1 to 6.0)**	-0.4 (-3.9 to 3.1)	0.62	6.7
	Model 2	Ref	1.6 (-1.7 to 5.0)	2.2 (-0.7 to 5.2)	-0.9 (-4.4 to 2.5)	0.98	5.6
State regulation	Crude	Ref	-1.9 (-4.7 to 0.9)	**-3.6 (-5.8 to -1.3)**	-2.5 (-5.0 to 0.0)	**<0.01**	28.9
	Model 1	Ref	-1.1 (-3.9 to 1.8)	**-3.5 (-5.9 to -1.8)**	-1.9 (-4.7 to 1.0)	0.05	15.6
	Model 2	Ref	-0.9 (-3.7 to 1.8)	**-2.8 (-5.3 to -0.3)**	-1.4 -4.3 to 1.4)	0.12	16.5
Autonomic stability	Crude	Ref	-0.1 (-1.1 to 0.9)	-0.7 (-1.5 to 0.1)	-0.2 (-1.1 to 0.7)	0.26	13.2
	Model 1	Ref	0.2 (-0.9 to 1.2)	-0.4 (-1.3 to 0.5)	0.2 (-0.9 to 1.3)	0.91	9.5
	Model 2	Ref	0.1 (-0.9 to 1.2)	-0.6 (-1.5 to 0.3)	0.1 (-1.0 to 1.1)	0.71	9.3
Abnormal reflex	Crude	Ref	-0.3 -2.2 to 1.7)	-1.5 (-3.0 to 0.1)	-0.5 (-2.2 to 1.3)	0.23	6.9
	Model 1	Ref	-0.2 (-2.3 to 2.0)	-1.1 (-2.9 to 0.8)	-0.0 (-2.2 to 2.1)	0.65	5.9
	Model 2	Ref	-0.2 -2.3 to 1.9)	-1.4 (-3.3 to 0.5)	-0.2 (-2.4 to 2.0)	0.51	5.6

Bold signifies p < 0.05.

Model 1 Adjusted for age of mother, maternal education, log income, maternal BMI, age at NBAS III assessment, parity, and birth weight.

Model 2 Adjusted for Model 1 plus Log As.

Table 5 shows the coefficients of the BSID II index scores at 6, 24 and 36 months after birth by caste group, again, with reference to *Brahmin*, the first-ranked caste group. In the crude model, *Vaishya* (the third-ranked caste group) showed higher MDI scores at six months of age (coefficient = 3.7; 95% CI = 1.3 to 6.0) than *Brahmin*. This association remained significant after adjustment for covariates (Model 1, coefficient = 4.0; 95% CI = 1.4 to 6.7) and covariates plus log-transformed As levels (Model 2, coefficient = 3.9; 95% CI = 1.2 to 6.7). However, the trend in MDI scores by caste at six months of age was not significant (*p* = 0.15 for Model 1 and *p* = 0.19 for Model 2). The remaining BSID II cluster scores were not associated with caste group at ages 6, 24, and 36 months.

**Table 5:** Coefficients of caste group on MDI, PDI and BRS scores of BSID II at 6, 24, and 36 months using multivariate regression model

			***Brahmin***	***Chetri***	***Vaishya***	***Shudra***	**p for trend**	**Constant**
			**(N = 37)**	**(N = 13)**	**(N = 26)**	**(N = 18)**		
6 months	MDI	Crude	Ref	1.4 (-1.6 to 4.3)	**3.7 (1.3 to 6.0)**	1.0 (-1.6 to 3.7)	0.07	103.1
(n = 94)		Model 1	Ref	2.1 (-0.9 to 5.1)	**4.0 (1.4 to 6.7)**	1.0 (-2.1 to 4.1)	0.15	118.4
		Model 2	Ref	2.1 (-0.9 to 5.1)	**3.9 (1.2 to 6.7)**	1.0 (-2.1 to 4.1)	0.19	118.5
	PDI	Crude	Ref	-0.5 (-6.6 to 5.5)	-0.9 (-5.7 to 4.0)	-0.4 (-5.8 to 5.0)	0.80	102.2
		Model 1	Ref	1.6 (-4.6 to 7.9)	1.1 (-4.4 to 6.6)	2.2 (-4.3 to 8.7)	0.52	123.0
		Model 2	Ref	1.6 (-4.6 to 7.9)	1.0 (-4.7 to 6.8)	2.2 (-4.4 to 8.8)	0.53	123.0
	BRS	Crude	Ref	6.2 (-3.4 to 15.9)	-0.1 (-7.8 to 7.6)	-4.0 (-12.6 to 4.6)	0.39	119.9
		Model 1	Ref	6.9 (-2.9 to 16.7)	4.5 (-4.1 to 13.2)	-0.2 (-10.3 to 10.0)	0.82	76.7
		Model 2	Ref	6.8 (-2.8 to 16.5)	2.9 (-5.9 to 11.7)	-1.1 (-11.3 to 9.0)	0.95	77.6
24 months	MDI	Crude	Ref	2.1 (-6.1 to 10.2)	-2.1 (-8.8 to 4.5)	-1.8 (-9.2 to 5.7)	0.49	91.6
(n = 89)		Model 1	Ref	4.6 (-2.9 to 12.2)	5.1 (-1.8 to 11.9)	4.4 (-3.7 to 12.4)	0.19	0.73
		Model 2	Ref	4.8 (-2.7 to 12.4)	6.3 (-0.7 to 13.3)	5.0 (-3.1 to 13.1)	0.13	-3.7
	PDI	Crude	Ref	4.0 (-5.5 to 13.6)	-2.5 (-10.3 to 5.3)	0.2 (-8.5 to 8.9)	0.75	103.3
		Model 1	Ref	6.4 (-3.0 to 15.7)	3.1 (-5.3 to 11.5)	4.2 (-5.8 to 14.1)	0.42	-184.5
		Model 2	Ref	6.3 (-3.0 to 15.7)	3.0 (-5.7 to 11.7)	4.1 (-5.9 to 14.2)	0.43	-184.4
	BRS	Crude	Ref	-0.5 (-11.5 to 10.5)	-0.6 (-9.6 to 8.4)	-4.7 (-14.8 to 5.3)	0.43	106.9
		Model 1	Ref	-0.3 (-11.7 to 11.1)	1.2 (-9.1 to 11.5)	-6.1 (-18.2 to 6.1)	0.49	-57.2
		Model 2	Ref	-0.4 (-11.8 to 11.0)	0.2 (-10.5 to 10.8)	-6.7 (-18.9 to 5.6)	0.39	-52.3
36 months	MDI	Crude	Ref	-1.4 (-7.9 to 5.2)	1.4 (-3.9 to 6.7)	-5.8 (-11.6 to 0.0)	0.20	97.4
(n = 83)		Model 1	Ref	-0.8 (-6.4 to 4.7)	3.0 (-1.8 to 7.9)	-1.9 (-8.0 to 4.1)	0.91	533.4
		Model 2	Ref	-0.8 (-6.4 to 4.5)	2.7 (-2.3 to 7.7)	-2.1 (-8.2 to 4.1)	0.98	538.5
	PDI	Crude	Ref	-4.3 (-10.5 to 1.8)	-0.6 (-5.6 to 4.4)	-2.0 (-7.5 to 3.5)	0.60	115.7
		Model 1	Ref	-3.6 (-9.0 to 1.9)	-0.5 (-5.2 to 4.3)	-1.4 (-7.3 to 4.6)	0.74	438.5
		Model 2	Ref	-3.5 (-8.9 to 1.8)	-1.5 (-6.3 to 3.3)	-1.8 (-7.6 to 4.0)	0.51	449.4
	BRS	Crude	Ref	-7.1 (-22.1 to 7.9)	-7.6 (-19.8 to 4.5)	-8.1 (-21.4 to 5.3)	0.17	105.7
		Model 1	Ref	-7.0 (-22.1 to 8.0)	-5.7 (-18.8 to 7.4)	-12.5 (-28.9 to 3.9)	0.15	43.0
		Model 2	Ref	-7.0 (-22.1 to 8.2)	-6.7 (-20.3 to 6.8)	-12.9 (-29.4 to 3.6)	0.12	54.0

Bold signifies p < 0.05.

MDI: mental development index, PDI: psychomotor development index, BRS: behavioral rating scale.

Model 1 of 6 months adjusted for baseline characteristics (maternal age, maternal education, log income, parity, maternal BMI, birth weight), age at BSID assessment on 6 months, weight of infants at 6 months, and HOME score at 6 months. For Model 1 of 24 months, baseline characteristics plus age at BSID assessment on 24 months, weight of infants at 24 months, and HOME score at 6 months were adjusted. For Model 1 of 36 months, baseline characteristics plus age at BSID assessment on 36 months, weight of infants at 36 months, and HOME score at 36 months were adjusted. Model 2 of all months adjusted Model 1 plus log As.

## Discussion

Caste was positively associated with one cluster of neurodevelopmental indicators at birth, namely state regulation as measured by the NBAS III. It was not, however, associated with BSID II scores at ages 6, 24, or 36 months, excluding MDI at 6 months. The positive association between caste group and state regulation at birth was partially mediated by cord blood As levels. Interestingly, the third-ranked caste group showed significantly higher MDI scores than the highest-ranked caste group.

To the best of our knowledge, this is the first study that has evaluated the effects of caste on neurodevelopment in younger children. In 1964, King Mahendra abolished caste system laws, declaring that as a nation Nepal opposed this form of population categorization. However, it is interesting that nearly 50 years after this abolition, the caste groups still show prominent association with neurodevelopment at birth. Thus, caste-related health disparities might still be prevalent in Nepal.

Similar to earlier studies [3-5,7,8,37-39], newborns in lower caste groups showed less optimal neurodevelopment at birth. This trend was partially mediated by cord blood As levels, suggesting that *in utero* exposure to As could drive the occurrence of lower-state regulation scores among lower-caste groups. The present study also showed that cord blood As levels were harmful to NBAS III state regulation cluster scores among the sample as a whole [26]. We also found that the *Vaishya* caste group showed higher cord blood levels of As than the *Brahmin* caste group. Since people from the *Vaishya* caste group are traditionally engaged in agricultural and outdoor activities, they may be more likely to be exposed to As.

The BSID II cluster scores did not differ across the caste groups at ages 6, 24, or 36 months. As suggested by Henn and colleagues, attenuation of this effect during the postnatal period may be one reason for this [40]. For example, urinary excretion of most of the As burden from the infant’s body may have occurred since decreased urinary As concentrations were also reported during the first four months after birth (80 μg/L during the first two days of life to <30 μg/L at four months of age) [41]. Hence, the harm induced by cord blood As levels on the neurodevelopment of infants might not persist until six months of age. Furthermore, the neuroplasticity of the immature brain may contribute to the attenuated effect of cord blood As over time [42].

Interestingly, infants from the *Vaishya* caste group achieved higher scores than those from the highest caste (*Brahmin*) on the MDI index of the BSID II scale at six months of age. Since those categorized as *Vaishya* members are merchants and agricultural workers, their infants may benefit from an environmentally enriched setting due to community visits to their houses and shops. Such an atmosphere might induce a level of neural stimulation etiologically relevant to neurodevelopment and learning, ultimately evident as higher MDI scores at six months of age. Alternatively, the higher MDI scores at six months of age among *Vaishya* members might be due to chance since we made 10 comparisons (7 from NBAS III and 3 from BSID II) that revealed a negative association between cord blood As levels and the NBAS III state regulation cluster. This negative association may be plausible because it was measured close to the time of birth.

The current study has some limitations, which should be considered. First, the small sample size and hospital-based sampling technique limit the generalizability of the findings. As such, associations alternative to those presented here may have been missed due to this lack of statistical power. Second, the classification of caste groups was based on family names. Although we can categorize caste precisely for *Brahmin* and *Chetri,* similar surnames and controversial classifications between *Vaishya* and *Shudra* might have caused occasional misclassification of caste groups. Third, postnatal As and Pb exposure was not measured, including levels in breast milk, drinking water, or other foods digested during the study period. Fourth, we did not assess breastfeeding status, although previous studies in Nepal reported that ever-breastfed rate was more than 99% regardless of SEP [43,44]. Fifth, additional confounds, such as history of asphyxia at birth, iodine status, and significant illness in early infancy, were not measured. Sixth, the Nepali translated version of the BSID II was not standardized in Nepal; thus, the findings in this study may not be generalizable to this specific region. Overall, this study has a number of strengths. The sample is the first longitudinal birth cohort from Nepal about which considerable information was collected regarding cord blood levels of toxic elements and potential confounds. Moreover, the NBAS III and BSID II were administered by a single investigator within the participants’ homes, increasing inter-rater reliability and diminishing underperformance effects by infants attributable to being assessed in an unfamiliar environment.

Collectively, these findings indicate that health education and awareness should be developed and implemented by local governmental health institutions among lower-ranked caste groups, especially among *Vaish*y*a* members, in order to educate them with regard to the detrimental effects of As exposure on neurodevelopment.

## Conclusions

Using a birth cohort study in Chitwan Valley, Nepal, revealed that caste was positively associated with NBAS III state regulation scores at birth, possibly mediated by cord blood As levels. Contrarily, BSID II scores at ages 6, 24, and 36 months were not associated with caste group. Finally, an inverse association between caste and MDI at six months was also observed. Further studies are needed to replicate the associations documented herein between caste and neurodevelopment at birth and their mediation by cord blood As levels. Most importantly, health policy recommendations should include measures to reduce exposure to As in Chitwan Valley, Nepal, especially among lower-ranked caste groups.

## Abbreviations

SEP: Socioeconomic position; Pb: Lead; As: Arsenic; EDTA: Ethylene diamine tetra-acetic acid; BMI: Body mass index; NBAS III: Neonatal behavioral assessment scale, third edition; BSID-II: Bayley scales of infant development, second edition; MDI: Mental development index; PDI: Psychomotor development index; BRS: Behavioral rating scale; HOME: Home observation for measurement of environment.

## Competing interests

We hereby disclose that the authors have no conflicts of interest, financial or otherwise.

## Authors’ contributions

RPP collected, analyzed, and interpreted the data and wrote the first draft; TF conceived study hypothesis, interpreted data, and finalized the manuscript; and MU and CW critically revised the manuscript. All authors read and approved the final manuscript.

## Pre-publication history

The pre-publication history for this paper can be accessed here:

http://www.biomedcentral.com/1471-2431/14/56/prepub
